# Voices and challenges of marginalized and vulnerable groups in urban informal settlements in Nairobi, Kenya: building on a spectrum of community-based participatory research approaches

**DOI:** 10.3389/fpubh.2023.1175326

**Published:** 2023-11-23

**Authors:** Robinson Karuga, Caroline Kabaria, Ivy Chumo, Linet Okoth, Inviolata Njoroge, Lilian Otiso, Nelly Muturi, Jiban Karki, Laura Dean, Rachel Tolhurst, Rosie Steege, Kim Ozano, Sally Theobald, Blessing Mberu

**Affiliations:** ^1^Department of Research and Strategic Information, LVCT Health, Nairobi, Kenya; ^2^Urbanization and Wellbeing Unit, African Population and Health Research Center, Nairobi, Kenya; ^3^Airbel Impact Lab, International Rescue Committee, Nairobi, Kenya; ^4^Department of International Public Health, Liverpool School of Tropical Medicine, Liverpool, United Kingdom; ^5^Faculty of Epidemiology and Population Health, London School of Hygiene and Tropical Medicine, London, United Kingdom; ^6^The SCL Agency, Wales, United Kingdom; ^7^Department of Demography and Population Studies, University of Witwatersrand, Johannesburg, South Africa

**Keywords:** CBPR, informal settlements, Kenya, marginalized, slums, urbanization, vulnerable

## Abstract

Urbanization is rapidly increasing across Africa, including in Nairobi, Kenya. Many people, recent migrants and long-term residents, live within dense and dynamic urban informal settlements. These contexts are fluid and heterogeneous, and deepening the understanding of how vulnerabilities and marginalization are experienced is important to inform pointed action, service delivery and policy priorities. The aim of this paper is to explore vulnerabilities and marginalization within Korogocho and Viwandani informal settlements in Nairobi and generate lessons on the value of a spectrum of community based participatory research approaches for understanding health and well-being needs and pinpointing appropriate interventions. In the exploratory stages of our ARISE consortium research, we worked with co-researchers to use the following methods: social mapping, governance diaries, and photo voice. Social mapping (including the use of Focus Group Discussions) identified key vulnerable groups: marginalized and precarious child heads of households (CHHs), Persons with disability who face multiple discrimination and health challenges, and often isolated older adults; and their priority needs, including health, education, water and sanitation. The governance diaries generated an understanding of the perceptions of the particularly vulnerable and marginalized informal settlement residents regarding the various people and institutions with the power to influence health and wellbeing; while photo voice highlighted the lived experiences of vulnerability and marginality. Understanding and responding to fluid and intersecting marginalities and vulnerabilities within growing urban informal settlements is particularly critical to achieving inclusive urbanization, where no one is left behind, a theme central to the Sustainable Development Goals and Kenya’s Vision 2030.

## Introduction

### Urbanization in Africa

High rates of urbanization have increasingly become a challenge for the vast majority of African governments and planners. Although Africa remains the least urbanized continent, it has the fastest urbanization rate in the world (1). The African Development Bank Group reports an average urbanization growth rate of 3.5 percent per year, which is expected to hold until 2050 (2). High rates of urbanization have resulted in the growth of informal settlements, with widening income inequities and rising urban poverty and hazards (1, 3, 4). Given the global estimates of informal settlement dwellers being at 1 billion people, urban inequities need to be addressed in order to support the goals of inclusive health and wellbeing for all as envisaged the Sustainable Development Goals (SDGs) (5).

### Vulnerability in informal settlements

Informal settlements typify urban exclusion and the socio-economic inequities that have characterized urbanization globally. However, too often residents of informal settlements are not represented in national statistics and face multiple access barriers to service provision, including for health and well-being. Beyond their numbers and spatial locations, the majority of informal settlement residents are poor (compared to non-informal settlement residents), manual, and informal workers (6–8). Residents of informal settlements face interconnected challenges such as rapid social, economic, demographic, and epidemiological transitions; multiple intersecting health risks and vulnerabilities; and complex, fluid governance arrangements, involving a mix of actors, with often longstanding neglect from state institutions (9). Vulnerabilities of people living in informal settlements are further compounded and clustered by social determinants including social marginalization, stigma, violence, alcohol and drug abuse, and fractured social structures. Further, risks and vulnerabilities, resilience, and response capacities are not evenly distributed, but influenced by intersecting inequities, including those shaped by socioeconomic status, gender, age, religion, disability, sexuality, ethnicity, location, employment, and citizenship (10).

Routine government data rarely cover urban inform al settlements and are usually insufficiently disaggregated. Thus, vulnerabilities and inequities are often invisible in urban planning, health and welfare information systems. Further, these vulnerabilities are poorly addressed in formal service provision. Consequently, the intractable challenge of conditions in urban informal settlements to date, has been linked to the poor conceptualization of the fluid and unequal social, political and economic relations that shape their governance, poor visibility of their health and living conditions to key decision-makers, exacerbated by their limited voices and power to demand redress. Addressing vulnerabilities in informal settlements requires new understandings of how to increase the visibility of residents living in informal settlements and their conditions and strengthen accountability arrangements for improving well-being and health, across a range of public and private, informal and formal actors.

In biomedical research, “vulnerable adults” are often defined as those who have a constrained ability to consent (such as people with cognitive disabilities) or those who are positioned as biologically vulnerable (such as pregnant women) (11). Conceptualizing vulnerability as a relational concept widens its potential scope significantly. Here, vulnerability is not a property of an individual but a relationship between individuals and others in specific times and places (11, 12). Vulnerability is most commonly created in relationships of unequal power and within wider systems of power and axes of inequity, such as social class, gender, caste, sexuality, age, disability, ethnicity, affluence and citizenship. We argue that vulnerabilities occur in situations of interpersonal power imbalances that are shaped by wider power structures. Indeed, hierarchies or differences in resources, are an important source of perceived inequalities, conflicts, and even wars (13).

Informal settlements are dynamic and encompass multiple and complex social, economic and political systems (14). Indeed, there is a growing awareness of this heterogeneity in the literature (15, 16). Informal settlements vary substantially in terms of population density, security of tenure, official recognition, provision of services, topography, and vulnerabilities including social and economic make-up (10, 17, 18). Informal settlements consist of many different groups with diverse interests, there are different degrees of infrastructure deficiencies and deprivation between and within informal settlement communities, with areas lacking secure access to tenure classified the same as an area lacking piped water, sanitation, and durable housing materials, leading to gradations in standards of living even within informal settlements, which amplifies health disparities (19). In many instances, the constant movement of people intensifies social fluidity and heterogeneity, which often result in the misleading framing of informal settlements as “ungoverned” spaces. Indeed, the near absence of formal government institutions creates multiple systems of informal governance, including community-based arrangements, non-governmental initiatives, private sector, and criminal organizations, as well as “clientelistic” relationships with political parties and state actors (20).

### The Kenyan context

In Kenya, despite informal settlement populations being highly mobile, about half of Nairobi’s population comprises relatively long-term dwellers who have lived in informal settlement settlements for over 10 years (8). While the Kenyan government has made efforts to reduce inequities in health outcomes at a national level, urban informal settlements and particularly vulnerable groups within them are being left behind (21). In Nairobi for example, many government initiatives to reduce health inequities do not effectively benefit the urban poor, despite their physical proximity to both public and private services (22–24).

From a population of 350,000 in the 1962 census to 4,397,073 in the 2019 census, Nairobi typifies the rapid urbanization and population explosion in sub-Saharan Africa (6). As the capital and largest city of Kenya, Nairobi has always been the major attraction of various segments of the Kenyan population—from rural and other urban areas—in search of better livelihood opportunities. The consequence of the rapid and uncontrolled population explosion is the proliferation of informal settlements in Nairobi, with upwards of 60 percent of Nairobi residents estimated to be living in informal settlements and contributing to increasing urbanization of poverty (25–27).

The search for pathways to reduce health inequities and improve health outcomes among populations in Kenya’s urban informal settlements, has identified the critical need for adequate data at the local levels. According to APHRC (2002, 2014), the importance of local informal settlement-specific data systems is reinforced by the limitations of global and national level datasets which generally produce national indicators that blur inter and intra sub-group inequities and often lack aggregation at local levels, where the needs are located. The limited coverage of informal settlement residents in nationally representative data samples means that existing national estimates do not sufficiently answer questions that are critical to the health and livelihoods of the urban poor, who constitute the majority of city dwellers (6, 22) Urban health programming is practically done at local levels by local governments, yet available evidence points to lack of the urban health statistics needed by implementing agencies and local governments, to measure progress, identify interventions that work or otherwise and pinpoint issues to prioritize in each informal settlement (28). The pursuit of a nuanced understanding of Kenya’s urban poor, calls for investment in evidence generation at local levels to enable contextualized policy and program interventions for specific informal settlements and sub-groups within them.

Making informal settlements and informal settlement populations visible has been recognized in recent years by a corpus of researchers as a worthy investment in the search for pathways to address urban deprivations, inequities and vulnerabilities (6, 10, 17, 22). Beyond surveys, there is a need for additional empirical methods that can explicitly highlight the levels and depths of marginalization and vulnerability of people who live and work in urban informal settlements.

Further, people living and working in informal spaces have “unsurpassed knowledge of relevant spatial and social infrastructures” (15) and so are in the best position to identify needs, priorities and actions to address health inequities in their environment. Therefore, there is a need for a co-creation process to collaboratively generate knowledge with people living and working in informal settlements to address visible and invisible structural, social and contextual barriers to health in informal settlements, including for water, sanitation, and hygiene (WASH), managing and controlling emergencies like the COVID-19 pandemic, developing infrastructure services and others (29–31).

### Community-based participatory research

Furthermore research is highlighting the need to move beyond a deficit model and recognize and support resilience in urban informal settlements (32). Community based participatory research (CBPR) and associated principles promote agency, power sharing, ownership and mutual sustained benefits for communities (33). CBPR approaches fall under the overarching definition of co-production research defined as a collaborative model of research that shares power between community partners, stakeholders and researchers working together to develop the agenda, design and implement the research, and interpret, disseminate, then implement the findings (34–36). Nonetheless, there is limited work that partners with, and prioritizes, the views and experiences of informal settlement residents, particularly those who are viewed as marginalized or vulnerable populations within urban informal settlements. CBPR is, therefore, a key methodological approach that can help these groups prioritize their health and wellbeing needs for action. This is critical to support understanding of nuanced vulnerabilities and lived experiences, and indeed the agency of informal settlement residents in demanding their rights to health (37).

This article is based on wider research in the “Accountability and Responsiveness in Informal Settlements for Equity” (ARISE) Research Consortium. The ARISE Consortium aims to increase accountability for marginalized people working and living in urban informal settlements to claim their rights to health across cities in Kenya, Sierra Leone, India, and Bangladesh (33). CBPR lies at the heart of the ARISE Consortium’s goal to catalyze a step change in approaches to improving accountability and promoting the wellbeing and health of urban marginalized people living and working in informal urban spaces. This article aims to explore vulnerabilities and inequities within urban informal settlements in Nairobi, Kenya and generate lessons on the value of community-based participatory research approaches in understanding health and well-being needs and appropriate interventions.

## Materials and methods

### Study sites

Our study covered two urban informal settlements- Korogocho and Viwandani- which are located between 7 and 12 km from Nairobi’s city center, and about 6 km from each other (38, 39). Residents of the two informal settlements present some differences with regards to population stability. Korogocho is more settled with multi-generational households as opposed to the youthful and highly mobile population in Viwandani. The two informal settlements are hosts to the Nairobi Urban Demographic and Health Surveillance System (NUHDSS) since 2002. These informal settlements are generally characterized by abject poverty, overcrowding and lack of access to water, as well as exposure to HIV/AIDS and sexually transmitted infections (10). Table 1 presents a summary of population characteristics in our study sites.

**Table 1:** List of marginalized and vulnerable groups identified by study community participants through participatory focus group discussions.

Vulnerable groups identified by study groups (order as prioritized)	Viwandani study groups	Korogocho study groups
Persons with disability (PWD)	Youth(F); Youth(M); Older adults (M); Older adults (F); CHV(M); FWM (M); Women 35–50 years; PWD (M); CBO (F)	PWD (M); PWD (F); Older adults (M); Sex workers (F); Men (35–50); Youth(F); Youth(M); VH (M); VH (F).
Older adults	Women 35–50 years; VH (M); CBO (M); Men 35–50 years;	CBO (F); Older adult (M)
Child–headed families Children (Orphans; street children; Boy-child and children from poor families)	SWM (M); CHV (F); Youth (F); VH (M); VH (F); FWM (M); CBO (F); Parent 8–12 years (F)	SWM (M); CHV (M); FWM (M); Sex workers (F); Women (35–50); Men (35–50); Youth (F); Youth (M); Parents-Adolescent (M)
Youth	VH (F); Youth (M)	Women (35–50 years) Youth (F)
Widows	PLWD (M); SWM (F)	VH (M); Older adult (F)
Poor/Jobless	Men 35–50 years; SWM (M); SWM (F); CBO (M);	PLWD (F); CHV (M); FWM (M);
Chronic Ill persons	Youth (M); Older adult (F); FWM (M); CHV (F); Refugees;	SWM (M)
Bad-behavior: witch, drug addicts	CHV (M)	Parents of Adolescent

F, Female; M, Male; CHV, Community health volunteer; FWM, Fecal waste management worker; SWM, Solid waste management worker; VH, Village head; CBO, Community based organization; PWD, Person with disability.

### Study participants, co-researchers and data collection procedures

This section describes the CBPR approaches we applied to recruit co-researchers, participants and explore their marginalization, vulnerabilities and associated inequities. We applied social mapping, governance diaries and photo voice. We defined co-researchers as equal research partners living in informal settlements and were directly impacted by vulnerability and marginalization. Co-researchers had an active role in the “research partnership” together with the researchers in this project. The partnership involved collecting data, co-analysis, and co-production of study interpretations. Participants were community members who took part in the research but were not directly involved in partnership with researchers.

### Social mapping

Social mapping is a process where community members are asked to illustrate diagrammatically what matters most to them. Between August and September 2020, we applied social mapping with community members in Korogocho and Viwandani to map the vulnerable and marginalized groups, drivers of marginality and vulnerability and responses to address both challenges in our study sites. Before data collection, we partnered with community advisory committee members as community mobilizers to sensitize our community-level participants about the study. Together with community groups, we convened interaction sessions and recruitment of participants and co-researchers in the communities. Using pre-tested topic guides, we explored perceptions about influential persons in the informal settlements, vulnerable and marginalized groups, social structures, and things one would change if they had power. A stakeholder analysis with the same community participants followed these discussions. The social mapping approach has been described in greater detail in another publication (51).

During the stakeholder analysis, community members in both study sites discussed who in their communities had the most and least influence over their living conditions. Community members also discussed and ranked the marginalized and vulnerable groups in the community, from the particularly marginalized and vulnerable to the least. Finally, our participants narrated the things they would change if they had the power starting from the most important to the least. Each social mapping exercise took approximately 45 min.

We triangulated the discussions on the vulnerable and marginalized groups using 10 focus group discussions (FGDs) with 80 participants who were part of the earlier social mapping. Each FGD took an average of 60 min. FGD participants were purposively recruited from all “villages” in the study sites (8 in Korogocho and 6 in Viwandani). FGD participants represented social groups such as: middle aged individuals, the older adults, youth, village heads, persons with disability (PWD), sanitation workers, garbage collectors, refugees, sex workers, parents of children, and community health promotors (CHPs). The design of our FGD topic guides was guided by the marginality framework, which clarifies the actors’ and agents’ roles in health and wellbeing, including the marginalized themselves. The marginality framework sees social and ecological systems as coupled, and facilitates the identification of the relationships between actors and public infrastructure (physical and social), and how the institutional environment creates structures that lead to processes of marginalization. The marginality framework draws attention to the marginality problem and thereby can facilitate targeting action on a set of root causes of marginality, such as social exclusion, ecological risks, or deficient institutions (40).

### Governance diaries

We employed governance diaries adapted from “financial diaries” (41) to understand the perceptions of marginalized informal settlement residents regarding various governance actors (people and institutions with the power to influence health and wellbeing) (41). Participants representing the particularly marginalized and vulnerable groups that were identified during the social mapping were recruited to participate. These participants represented children heading households (CHHs), persons with disability (PWD), and older adults. The process included in-depth interviews with 24 participants (4 CHHs, 4 PWD, and 4 older adults in each of the two study sites), participant written diaries, two local level consultative meetings in each study site, participant observation with resulting reflection diaries, photographs and informal discussions. All of these aimed at understanding the realities of the different contexts. We recruited community co-researchers who were based in the study sites to participate in data collection and co-analysis.

Further, there were individual, team, mixed group, and researcher-co-researcher reflexivity sessions for cross learning and to deliberate upon upcoming issues on a weekly and sometimes biweekly basis. The continuous presence of researchers in the field sites for 4 months in each site facilitated rich descriptions of social activities, analysis of governance actors and actions. For this study, we collected data for 4 months with six visits to the study participants’ residences. The first meeting enabled the study team to identify the “authorities” around the participants and the subsequent data collection sessions enabled the team to collect data on how “authority” affects their health and wellbeing and also identify other emerging problems. Diaries also allowed for confidentiality and unguarded responses that are not possible with face-to-face interviews. The participants were provided with attractive diaries, with guidelines pasted in the front of the diary. Each participant wrote their daily activities related to governance on six priority themes (education, water, sanitation, health, hygiene and solid waste management) to the community, without writing their names in the diary. Community co-researchers supported participants using phone calls and impromptu visits. Co-researchers also reminded participants to complete their diary entries during the week. During the six rounds of visits to conduct in-depth interviews with informal discussions and observation of priority areas, participants reflected on why they chose the entries in the diaries, how it made them feel, what they learned and the implications for current and future governance. We encouraged participants to view governance diaries as their personal journals. A detailed description of how the governance diaries methods and interpretation of findings has been published elsewhere (52, 53).

### Photo-voice

We further implemented the participatory wellbeing and governance analysis and priority setting using the photo voice approach. Photo voice is a participatory visual method that provides a platform for marginalized and vulnerable social groups to share their lived experiences using photography. In this CBPR approach, we issued smart phones to participants to enable them to act as “co-researchers” to identify and reflect on the daily lived experiences (42, 43). Photo voice allowed us to explore and understand the persistent problems in the study settings in a way that was most relevant to them (44). Before we applied photo voice, we created awareness on the study among community leaders and gate-keepers. Our awareness creation meetings involved sharing clear information to the community gatekeepers about our study objectives and utility. After receiving clearance from the community gate keepers, we partnered with them (clergy, elders and community health volunteers) to purposively select participants.

We purposively sampled 19 participants that represented the vulnerable and marginalized in both study sites, as identified by the social mapping reported above. We requested for written consent from all our participants before the study, after which we trained them for 2 days on how to take photos that conveyed meaning and photography ethics using simple terms and definitions. We provided our participants adequate internet bundles and airtime throughout the study by regularly topping up their internet credit and airtime. We activated the smart phones to synchronize photographs into cloud storage to ensure real-time backing-up of photographs. We installed the WhatsApp^™^ application to facilitate sharing of photos with the research team daily. Photos shared via WhatsApp^™^ were backed up in a password-protected cloud database. This process has been described in another publication (21).

We conducted the photo voice process in three stages, over a three-week period. During stage one, we asked participants to take photos that reflected their day-to-day life, highlighting what marginalization meant to them and how it was reflected in their daily lives. In stage two, we asked participants to take photos that reflected what health and well-being meant to them. In stage three, we asked participants to take photos that depicted what needed to be addressed to improve their health and wellbeing. Researchers conducted face-to-face in-depth interviews with the study participants to explore the motivations for taking each photograph. During the weekly IDIs, we requested each participant to choose five (5) photographs that would be shared with other participants and discussed during a co-analysis workshop. Our participants came up with captions that described each of the five selected photographs using their own words. We obtained additional written consent to use their photos for advocacy and publications.

We conducted additional in-depth interviews with 12 additional marginalized participants, who were not part of photo voice. These additional IDIs explored experiences and perceptions of marginalization and vulnerability, and what can be done to alleviate these experiences. We conducted 6 focus group discussions (FGDs) with governance actors (village elders, *Nyumba Kumi* leaders (community household units’ leaders) CHVs, women and youth leaders and religious leaders) to understand existing informal and formal governance structures and their areas of influence. FGDs also explored accountability mechanisms, related challenges and possible recommendations.

The foregoing three methodological approaches and their different elements are summarily presented in Table 2.

**Table 2:** Summary of community based participatory methods that were implemented in the study sites.

Methods	Purpose of method	Description of use and roles	Study participants
Social mapping/charting FGDs	To identify the key vulnerable and marginalized groups in the population. To identify the drivers of marginality and response to marginality.	Being local themselves, the co-researchers played the major role in accessing the community. Used to get the deeper insides of the vulnerable and marginalized groups. Charting/mapping themes (stakeholders, influential groups, marginalized groups, vulnerable groups, social structures, and things one would change if they had power).	Purposively selected people living in the study sites. FGD with 80 participants represented people from each study villages, including older adult, middle aged people, youth, village heads, PWDs, sanitation workers, CHVs, parents of children, refugees, and sex workers.
Governance Diaries	To understand the perceptions of the vulnerable and marginalized groups toward the governance stakeholders. To understand how vulnerable and marginalized groups navigate around governance.	Participants wrote their diaries reflecting their experience of accessing any governance services. Consultation meetings and informal discussions to understand the realities of different context. Reflexivity sessions held with the presence of researchers for a longer period helped to get richer descriptions and understanding of governance aspects in the informal settlements. Each participant would write their daily experience around governance activities related to education, water, sanitation, health, hygiene and solid waste management	24 persons representing marginalized and vulnerable groups identified from the social mapping exercise above, i.e., PWDs, heads of child headed households and elderlies, 4 each from each category from each of two study sites. Reflectivity sessions with individuals, group, mixed group and co-researchers / researchers.
Photo-voice IDIs FGDs	To analyze well-being and marginalization with informal settlement residents using participatory approaches. To record and reflect on day to day lived experience on marginalization. To understand the existing informal and formal governance structures and their areas of influence.	Photo voice enabled an in-depth understanding of the day-to-day lived experiences of marginalization that affect the health and wellbeing of vulnerable and marginalized groups.	Data were collected from a total of 37 persons in the study sites (19 participants represented the three marginalized groups, 12 residents were purposively selected from the study sites, and 6 were governance actors in the study sites).
Stakeholder analysis	Ranking of the stakeholders (lowest to highest influence). Ranking of the vulnerable and marginalized groups (particularly marginalized/vulnerable to least)	The community people ranked the stakeholders from lowest to highest influence in the life of people living in the informal settlement. Similarly, vulnerable and marginalized groups were also ranked based on their vulnerability.	We purposively selected 80 persons who lived in the study sites to participate in the stakeholder analysis.

### Data analysis

We conducted the analysis in two stages. First, five community co-researchers who were involved in social mapping and completing governance diaries were invited by the researchers to participate in developing the coding framework. Community co-researchers read the draft coding framework independently and suggested modifications and new themes that may not have been considered (52, 53). Secondly, we organized a photo exhibition with the photos that had been prioritized by the marginalized and vulnerable participants. We then asked community co-researchers to suggest themes based on their interpretation of the displayed photos. The emerging themes from this co-analysis stage were added into the coding framework.

Stage two of the analysis involved the research team. To ensure that no information was missed, researchers analyzed the data as it was being collected. Audio recordings from IDIs and FGDs were transcribed into MS Word^™^ and crosschecked by a third party to ensure all the information was captured in the transcripts. The transcripts were then translated to English (where necessary) and cross-checked to ensure that the translation did not alter the meanings of the data. The transcripts were uploaded into NVIVO 12 software (QSR International, Australia) for coding and analysis. The transcripts were then analyzed using the content analysis approach. This involved reading the transcripts multiple times to gain a sense of the flow of the discussion. Thereafter, the transcripts were coded following emergent codes such as particularly vulnerable groups, drivers of marginalization, experiences of marginalization, and response to marginalization among other themes. This coding was applied to all transcripts and as new codes emerged, they were added to the list of codes and the transcripts read again to ensure that all the transcripts had been coded adequately. Codes that were similar were combined into single categories through consensus discussions. The next step entailed producing “tree nodes” or major categories that were inductively synthesized from the previous steps. The themes identified formed the basis for providing a robust picture vulnerable and marginalized groups in the informal settlements as well as identifying the most vulnerable and marginalized groups across the settlements. We convened reflexivity meetings between research team members and co-researchers to reflect on our values, cultures, prejudices and how these influenced the way we interpreted the data and perceived our participants. After the preliminary analysis, we conducted a series of member check in workshops to allow community leaders, study participants and governance actors to validate these experiences of marginalization and vulnerability (Tables 3, 4).

**Table 3:** Characteristics of photo voice study participants.

Persons with Disability	Type of study participant	Axis of vulnerability
1. Older adults (*n* = 7)		
Male PWD – one hand	Photovoice participant	PWD – one functional hand, male caregiver for his three primary school-going children, low social-economic status, and alcoholic
Female PWD – Deaf	Photovoice participant	PWD (deaf), low social economic status, no source of income, older adult, lives with a husband who is also deaf and with no source of income.
Male PWD – Blind	Community IDI interview	PWD (blind), low social economic status, no source of income, and poor housing structure
Female PWD – Physical	Community IDI interview	PWD – (physical disability) – moves with crutches, low social economic status, failing business, and lives by herself
Male PWD – Physical	Community IDI interview	PWD – (physical–on a wheelchair), low social economic status, no source of income and lives with his brother who scavenges at the damp site
Male PWD – Physical	Community IDI interview	PWD – (physical, was run over by a vehicle), low social economic status, no source of income, abandoned by wife after an accident that crippled him, and suicidal
Female PWD – physical	Community IDI interview	PWD – (physical), low social economic status, no regular source of income and the caretake of two of her children.
2. Children heading households (*n* = 6)		
Female	Photovoice participant	Young girl (17 years), school going, low social economic status, primary caregiver for a family, sick mother, many dependents (alcoholic sisters, nieces and nephews), two sisters died following each other during the study period.
Male	Photovoice participant	Young boy (17 years), school going, low social economic status, disabled mother, other dependents, and primary caregiver for a family
Male CHH-R1	Community IDI interview	Young boy (17 years), School drop-out, low social economic status, away from the biological mother, alcoholic father, and stepmother, support his 5 siblings and mother back in the rural home
Male CHH-R2	Community IDI interview	Below 18 years, orphan, School dropout (primary level)low social economic status, lives by himself and no social support by family, poor housing and environment
Female CHH-R3	Community IDI interview	She is a teenage mother below 18 years, has undergone GBV, was abandoned by the mother, low social economic status did not proceed to secondary school, head of household depended upon by 3 siblings, poor housing, No income and food insecure
Female CHH-R4	Community IDI interview	Low social economic status, no income and food insecurity, abandoned by the mother, did not proceed to secondary school, head of household and depended upon by 2 siblings
3. Older adults (*n* = 6)		
65-year-old Male	Photovoice participant	Older adult (above 65 years), sick and deteriorating health due to a growth on his face and general fatigue, low social economic status, suffered catastrophe (fire razed down his residence & rentals), lived by himself. Non-supportive family back at home, his family depended on him, disappointed by his family his son, and his daughter. Son was gunned down by police, wife does not care for him and sides with sons. Zack died just before the end of data collection. He was found in his house dead.
65 year old, Female	Photovoice participant	Older adult (above 65 years), female, sick and deteriorating health (lives with diabetes), low social economic status, no source of income, has no support system – son mentally ill and dependent on her, poor housing.
63-year-old, Male, ED-R1	Community IDI interview	Older adult (63 years), a male who lives by himself, low social economic status, no source of income, poor housing – house affected by air population (smoke and smell from the damp site)
77-year-old, Male, ED-R2	Community IDI interview	Older adult (77 years), low social economic status, no source of income, and seemed a very bitter person
65-year-old, Female, ED-R3	Community IDI interview	Older adult (above 65 years), sick (diabetes and high blood pressure), low social economic status, no source of income, and fends for self
65-year-old, Female, ED-R4	Community IDI interview	Older adult (above 65 years), low social economic status, poor housing affected by air pollution from brewers, depends on self and caring for grandchildren

**Table 4:** Summary of population characteristics of Korogocho and Viwandani informal settlements (54).

	Korogocho	Viwandani
Geographic area	0.9km^2^	5km^2^
Total Population	36,900	43,070
Total Number Households	11,757	18,472
Total Number of Children under 5	3,642	6,361
Dependency ratio* (%)	0.59	0.41
Currently Married Residents aged 15–49 years		
Females (%)	38.2%	43.9%
Males (%)	40.5%	48.2%
Secondary+ Educated Residents aged 18+ years (at least 12 years of school)		
Females (%)	35.6%	47.5%
Males (%)	41%	54.7%
Unemployed Residents aged 18+ years		
Females (%)	41.6%	33.9%
Males (%)	19.7%	8.1%

## Results

The results draw findings from across the various CBPR methods to give a holistic understanding of each group’s unique challenges. Using social maps and FGDs, different community groups identified children heading households (CHH), persons with disability (PWD), and older adults as the particularly marginalized and vulnerable social groups within their communities. Governance diaries provided an opportunity to build long term relationships and understand temporal aspects of governance as it relates to the marginalized groups. Finally, photo voice gave a unique visual insight into the daily lives and lived experiences of these groups through their own understanding. The unique challenges and social inequalities, and their implications for access to health and wellbeing services, these groups face are highlighted in the results. We present these by key themes that shape vulnerability and marginalization.

### Livelihood opportunities insufficient to meet basic needs

Lack of opportunities to participate in livelihood activities that were sufficient to meet their basic needs was a common key feature of vulnerability for all groups in both communities. Lack of opportunities for persons with disability and older adults related to their physical challenges to participate in the available livelihood activities. In contrast to rural settings older adults lack traditional family support and financial resources in a context where they are increasingly unable to earn a daily livelihood due to physical and/or mental infirmity.

*“Older adults are marginalized…from sixty-five {65 years} going up, those who cannot help themselves unlike in most rural settings where there is communal support. There are those in these ages {65+} and have no one to help them, they are just there without basic needs…”* (FGD, CBO, Viwandani, M).

Children heading households faced challenges accessing work due to their lack of opportunities to develop skills and lack of social and monetary capital. They were also left vulnerable to exploitation by informal employers and their involvement in income generation reduced their capacities to participate in education with likely long-term impacts on their socio-economic status.

**Figure 1:**
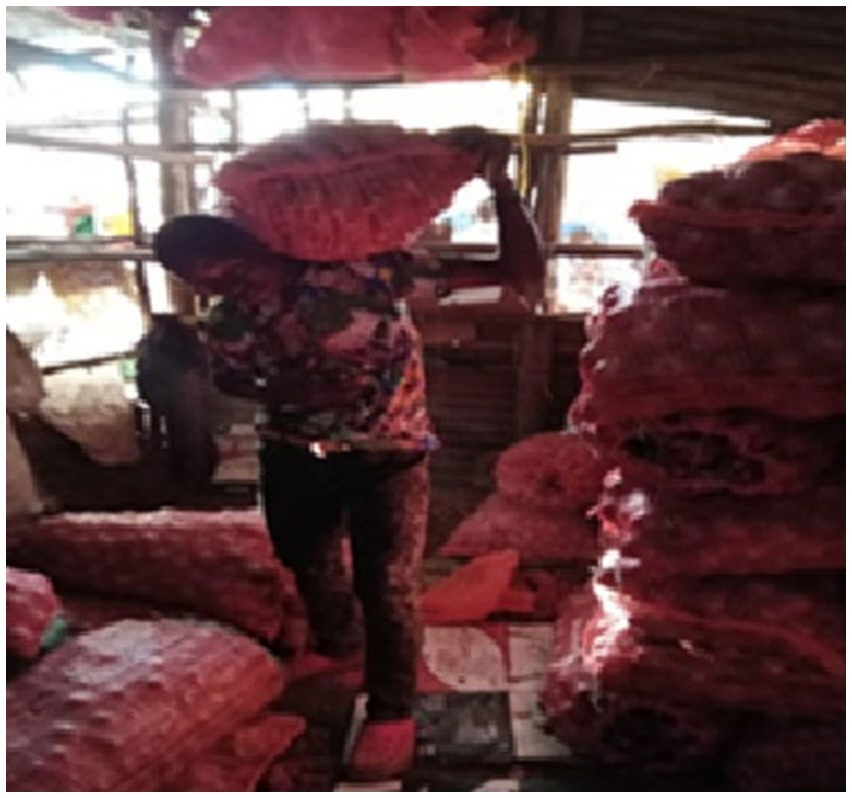
Photo depicting exploitation of children heading households by local community members.

Children heading households were vulnerable to exploitation. Some residents engaged these children to work for long hours with no or little pay. One participant narrated how he was engaged to do hard casual job for a merchant at a local market, and got paid KES 300 (Approx. USD 2) per day. Figure 1 was taken by a child depicts this theme.

*“When I have done a lot of work by the end of the day, he gives me three hundred shillings …I feel very sad because I picture myself as an employer and not employee especially doing odd jobs for a living. I wonder for how long I have to rely on other people to employ me for a living”* (IDI, Child heading household, Korogocho, M).

**Figure 2:**
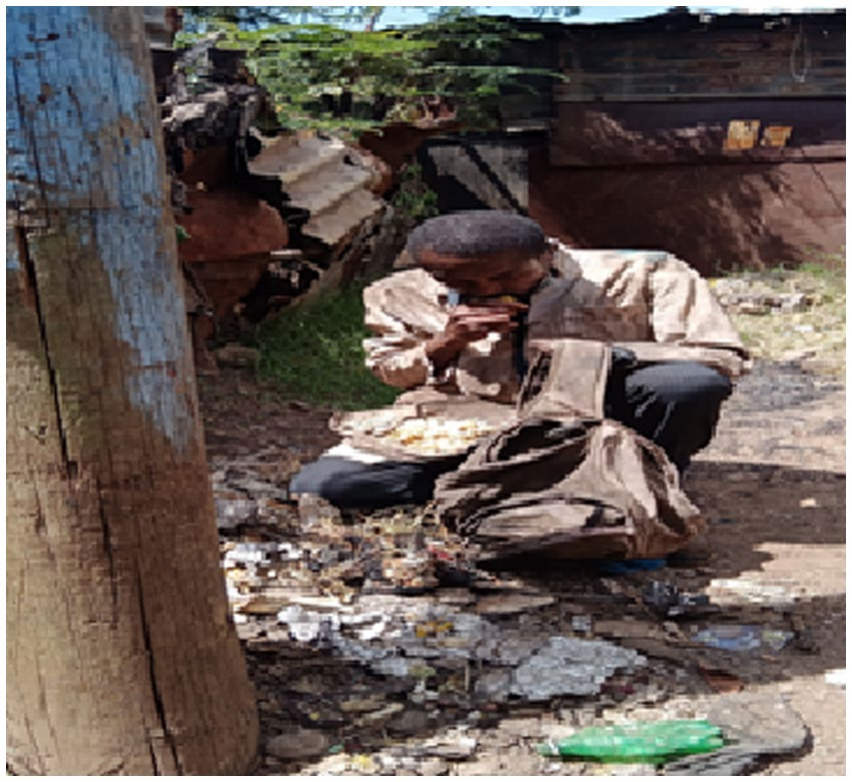
Participant with disability resorted to scavenge for food to sustain himself and his family.

Participants from all three groups often resorted to scavenging through the dumpsite for recyclables to sell to provide food for their families or directly scavenging discarded food. This was reported to be a way to provide for children and for persons with disability. Figure 2 was taken by a participant who is living with disability to reveal how he resorted to scavenging for food.

“In this photo am wearing gwanda [the clothes that I use to work]. I usually have two bags one for food and the other one for carrying recycling waste materials. The garbage collected outside residential homes in garbage bags guarantees a lot of food. If you search inside, you cannot fail to get plastic materials like jerry cans and so on. You will also find food like bread and that is why I have to wake up very early in the morning so that I find it. This affects me because I do not feel happy eating that food, but I have to because the money I get cannot sustain all my needs at home” (IDI, Person with Disability, Korogocho, M).

**Figure 3:**
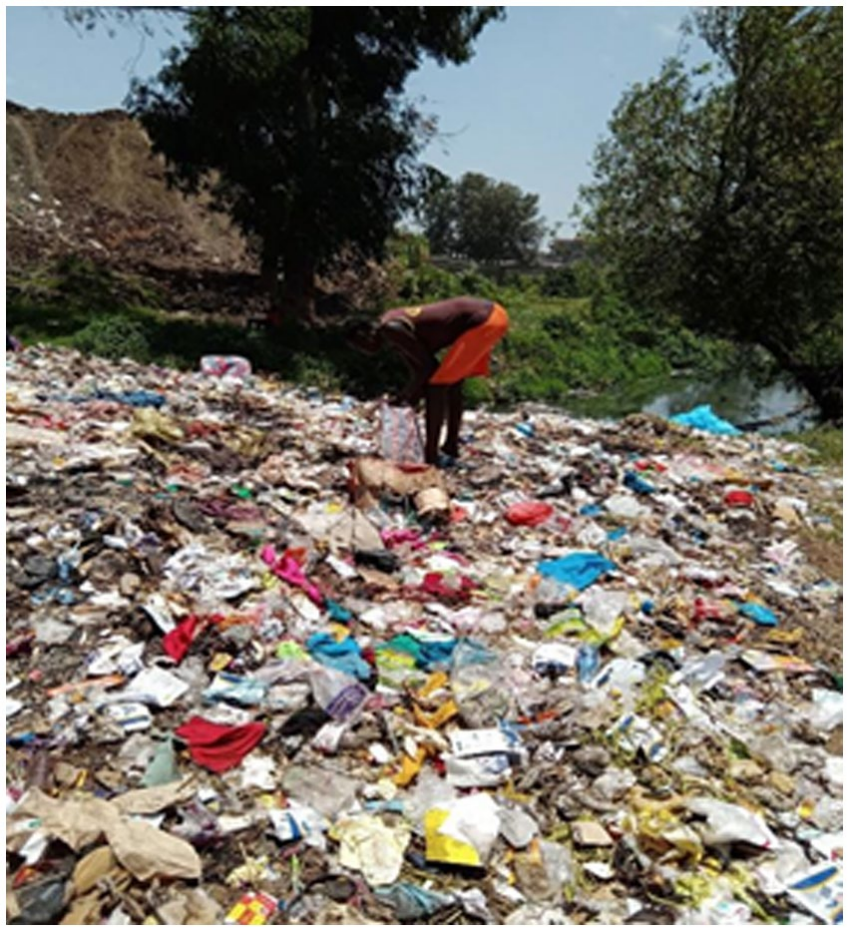
Female child head of a household scavenging in a waste dumpsite (Taken by a child heading a household).

The COVID-19 pandemic exacerbated the experiences of marginalization and vulnerability of children heading households because they could not generate enough income to feed their families. With reduced work opportunities and school shut down which prevented accessing food through school meals, these children had to scavenge in the dumpsite, which exposed them to other health and safety hazards (Figure 3).

All participants described food insecurity, including prolonged periods without food, or being able to afford only the cheapest and least nutritious food such as *ugali*. The COVID-19 pandemic further exacerbated the experiences of marginalization and vulnerability for all participants children heading households since income generating opportunities were reduced throughout the communities, increasing competition for the limited available opportunities as narrated by one child.

*“Yes right now it is very difficult to get someone who will accept to give you work. Mostly it is only the dump site where you can go and get something that can earn your money because at the dump site there are no restrictions on COVID-19. At the dump site people are just living as usual but in other places like where the Somalis stay, if you go right they will not accept you to get in because they say that you infect them with corona* [referring to COVID 19]” (IDI, Child heading household, Korogocho, F).

For children heading households, school shutdowns also prevented them accessing food through school meals. Older participants reported being unable to adhere to medication due to lack of food, and also poor access to water due to lack of money and also lack of physical strength to carry water to their households.

### Burden of care and poor access to services within households

Both older adults and children heading households narrated the challenges they faced due to having to care for dependents, including younger siblings and disabled parents for child heads of household, and both adult children and grandchildren for older adults. The burden of care included taking responsibility for providing food, school fees, and other basic needs as well as reproductive work such as washing as narrated by two children:

*“The first one would be my mom. She is not able to go to work which means that sometimes I have to miss school to be able to provide for my family, pay rent”* (IDI, Child heading household, Korogocho, M).

Another child narrated:

*“There are times when I miss going to school so that I can go to work… because I.*

*have to go to work so that I can get money to feed my mum and younger siblings because if I do not do that there is nobody else to do it”* (IDI, Child heading household, Korogocho, F).

Persons with disability faced the contrasting challenge of lack of support and care from other family members. Common factors in shaping households without support from working age adults were disabilities, chronic disease and mental health issues, including drug and alcohol dependence, highlighting the significant burden of unmet health needs within these communities. Poor access to healthcare was narrated and documented by participants from all three groups and a common feature of this was insufficient money to pay for medicines, which were generally unavailable at public health facilities. Persons with disability additionally narrated the lack of adaptations to service provision such as sign language interpretation which would enable them to access services. Older people narrated the mental health burden of being unable to afford to access services for which they had been referred for worsening conditions. Older adults were often in a position of having younger dependents, so even those who do receive some form of social security such as cash transfers, this was often required to support them, including school fees.

*“I stay in this house with my grandchildren who are schooling and have no one to help me to access basic services. For the cash transfers, I end up using it for the needs of dependents who do not have any means of livelihood”* (IDI, Older adult, Korogocho, M).

*“Those who receive cash transfers end up using it for the needs of dependents who do not have any means of livelihood”* (FGD, CBO member, Viwandani, M).

### Social stigma and discrimination

Participants from all groups narrated experiences of social stigma and discrimination within their communities, which intersected with their (lack of) livelihood opportunities, poor access to services and their family situations to worsen their sense of isolation and poor mental health and social wellbeing as narrated by one participant:

*“I feel like I am marginalized because when I go out to search for work, when people see that I have one hand they would just keep on postponing you by saying ‘you come tomorrow the day after tomorrow but that tomorrow will never reach’. So, you end up wasting time hoping that you will get that job so that you can be able to provide for the children at home. It would have been better if this person could have given me that job and I fail to do it instead of lying to me to come back the following day all the time.”*(IDI, Person living with disability, Korogocho, M).

Contrary to a cultural tradition of respect for older adults were described as often left out of community discussions, decision-making and access to information and new knowledge, and left feeling physically and emotionally isolated.

*“We are left out in community activities and decision making… even community education nobody remembers the older adults”* (FGD, Older adult, Korogocho, M).

*“Because of my old age, no one thinks that I am worthy to be taught anything new or to receive any support”* (IDI, Older adult, Viwandani, F).

Persons with disability described how they were often discriminated against and excluded from development activities, often due to cultural and traditional beliefs and had limited access to services and reduced livelihood opportunities. Participants identified persons with mental health conditions, amputees, and visually impaired as particularly marginalized due to discriminatory and stigmatizing perceptions of people with mental illness. Participants described how PWDs were often hidden away and therefore not included in the provision of basic services.

*“The mentally ill…are also marginalized. When they talk something which is right; it appears that they are wrong because they are mentally ill”* (FGD, CBO, Viwandani, F).

*“The disabled are marginalized…An individual has a disabled person in the house but they hide them, they do not want them to be seen. When you go in and check at this child…the way they are taken care of is not the same as the rest of the children…They do not often bath them or feed them if they are unable to feed themselves.”* (FGD, CBO, Viwandani, F).

Child heads of households reported enacted stigma by peers based on their scavenging livelihoods and poor living conditions and documented in the paper by Karuga et al. (21).

### The nature and impact of the built and physical environment on health and wellbeing

Participants from all groups narrated how on how the built and physical environment including, housing conditions, drainage, road infrastructure, sanitation/toilets and solid waste disposal, impacted their health and wellbeing vulnerabilities. For example walkways with open drainages were occupational hazards for PWD, children heading households (21), and older adults, especially when it rained. Roads paved without pedestrian walkways and covered drainages also posed serious movement challenges for PWDs.

*“The visually impaired people are in difficult situation because they have so much challenges they can hit something or fall in a hole and no one will help them out because they have disability… Even if I have disability I use crutches and keep going unlike the blind, so the blind, deaf and the paralyzed are more marginalized and vulnerable.”* (FGD, PWD, Korogocho, M).

**Figure 4:**
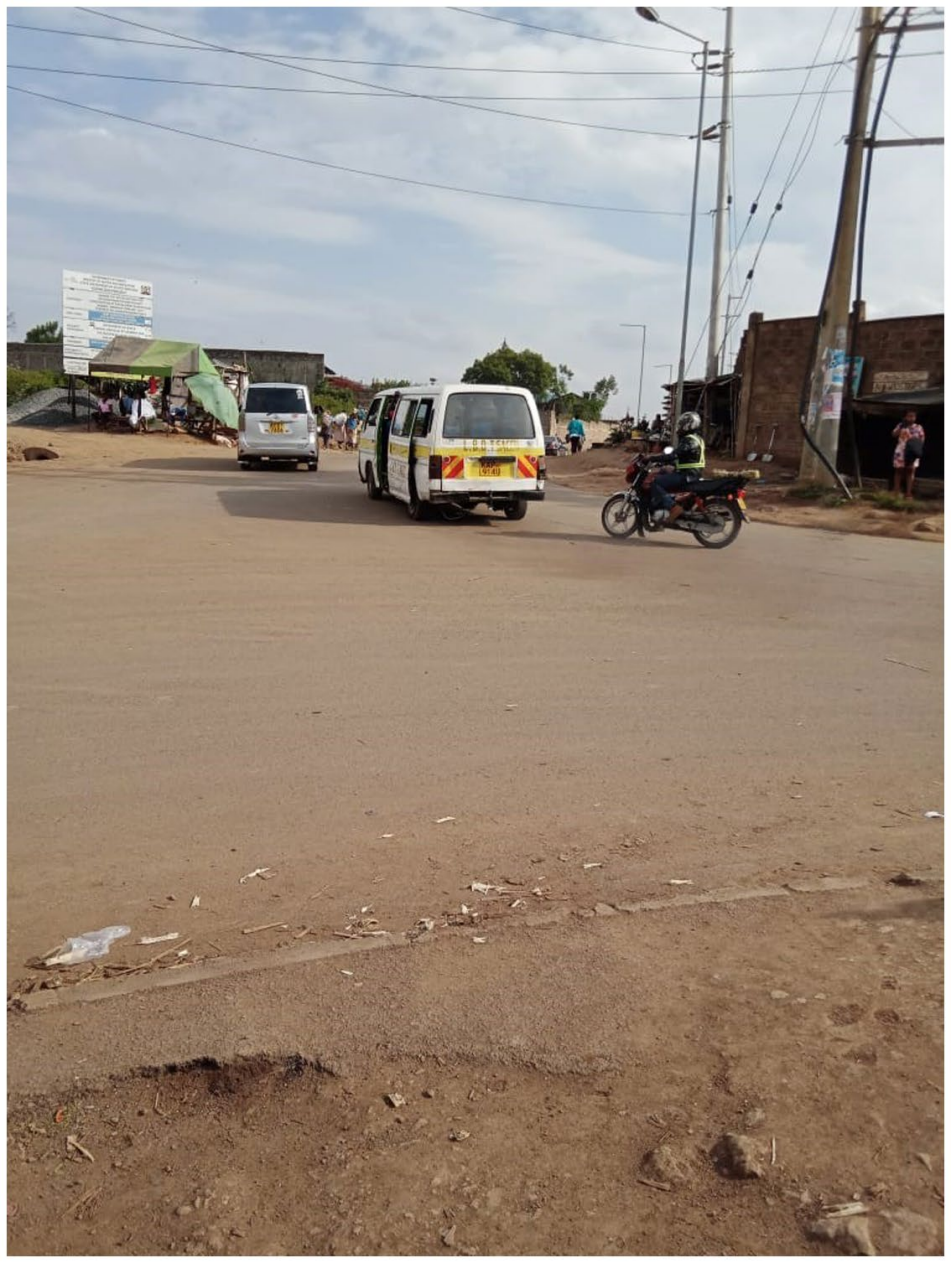
Photo of the built environment: roads without signposts, markings, and speed controls as well as and drainages without covers (Taken by a PWD).

These also constituted serious health hazards including road traffic accidents as road users are not conscious that there are PWD crossing the roads (particularly the deaf who would not hear any warnings of oncoming traffic characterized by loud noises of oncoming vehicles or hooting). When roads are improved without markings as in Figure 4, drivers often maintain a good speed oblivious of the slow pace of road users with disability and older adults, often resulting in fatal accidents.

Infrastructure in urban informal settlements falls short of meeting the requirements of many older adults. Inadequate access to water and essential sanitation is a source of hardship for older adults. All older adults mentioned inadequate infrastructure for water supply as a major source of marginalization that affected their health and well-being. Older adults are not able to walk long distances to fetch water. Most older adults had to pay younger residents to carry water for them into their homes, as stated by a female participant as she described photo that she took:

*If you look at the picture I took, you can see the commotion during fetching water… I have to look for someone to do it on my behalf and some would ask for money which I do not have sometimes. If I do not have money I have to rely on a kind person who will help me carry it, the young energetic young men will always ask for money. I have to wait in the queue at times until someone volunteers to help me carry it and take it home. That weighs me down so much and my wish would be that the government provides water.* (Older female participant, Korogocho).

**Figure 5:**
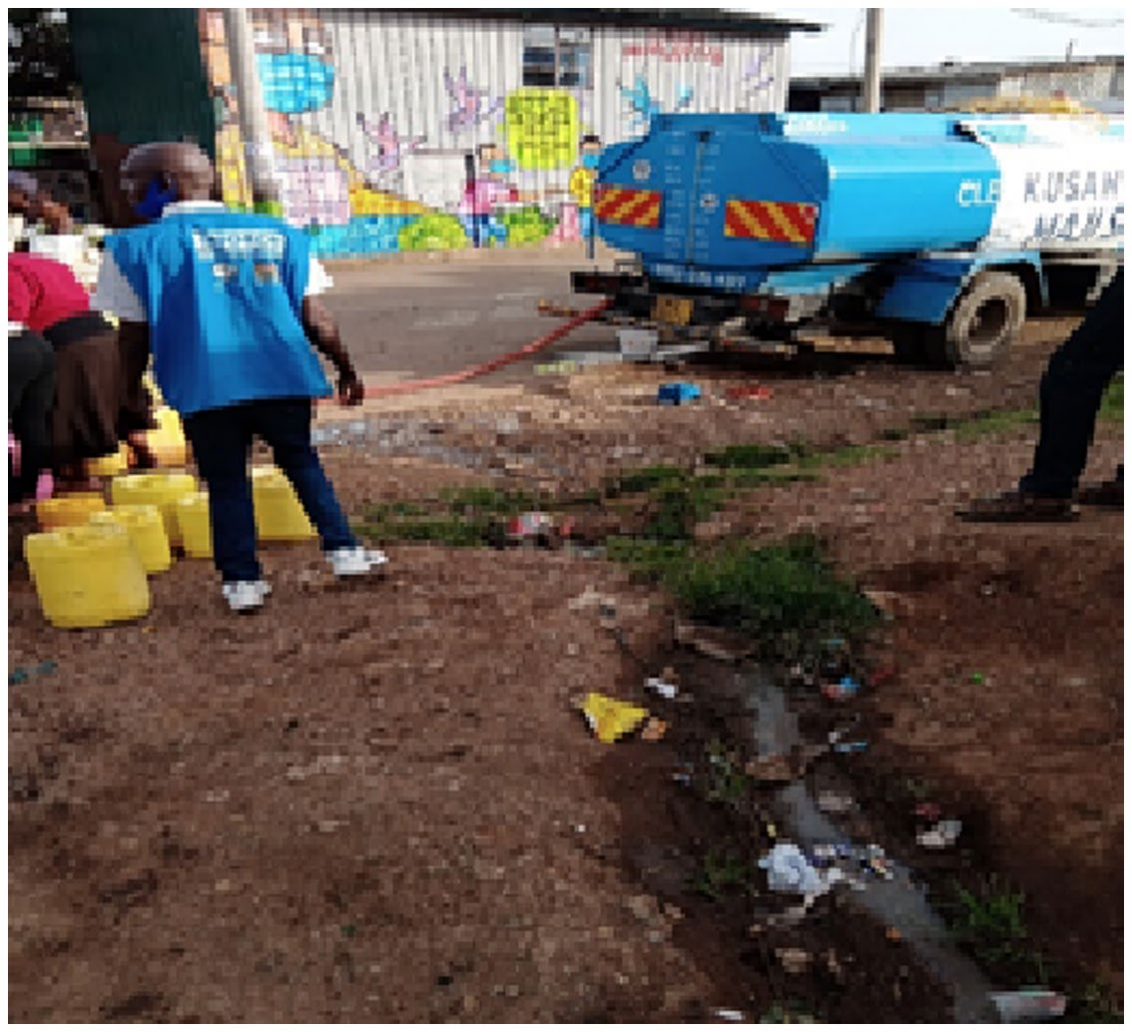
Residents jostle for water distributed free of charge.

**Figure 6:**
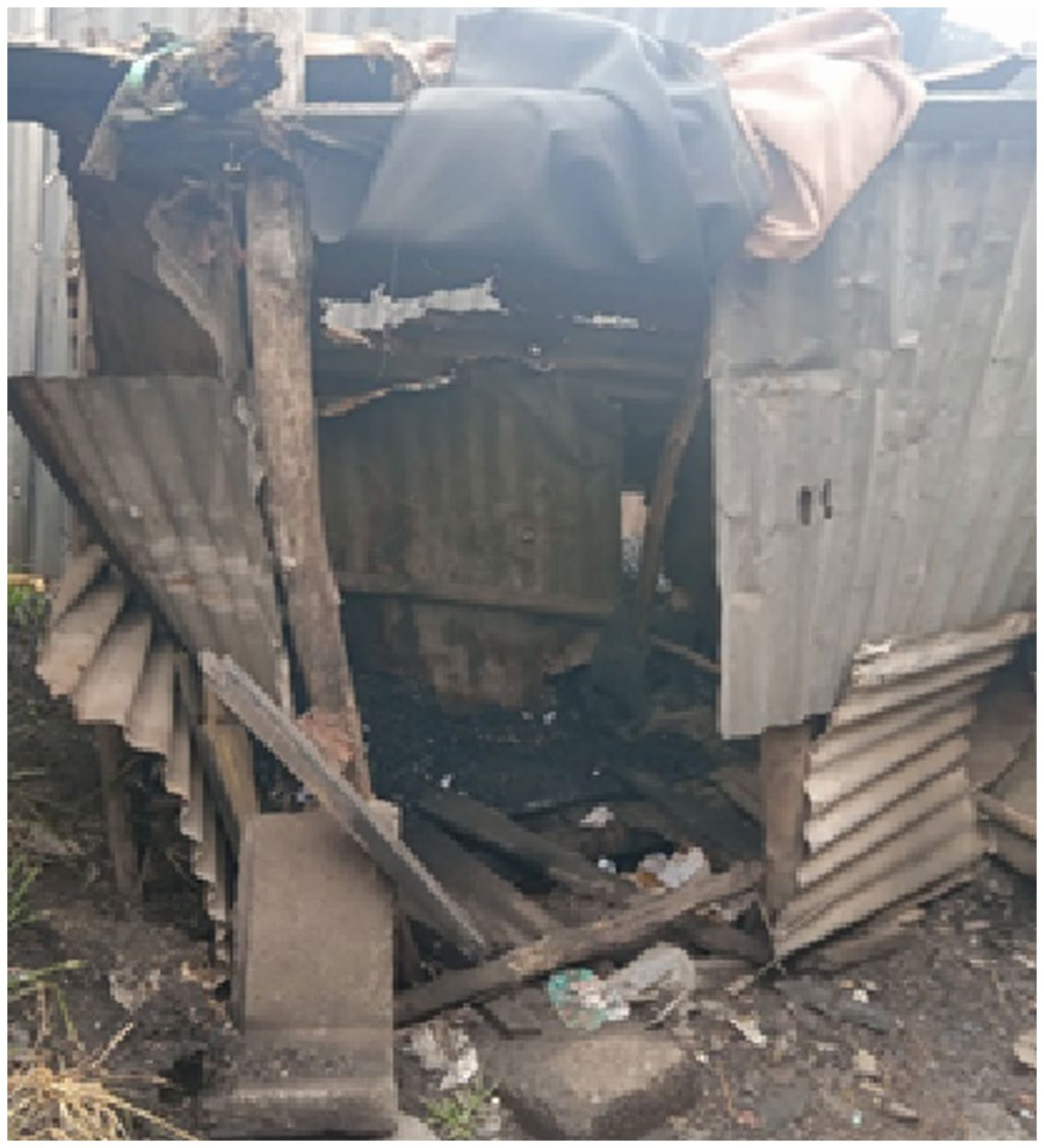
Photo representing marginalization of older adults in access to toilet facilities in informal settlements. Taken by a female older adult.

Older adults narrated that houses in the urban informal settlements are built without toilets hence. They have strained to access toilet facilities resulting in use of paper bags to relieve oneself, especially at night. Available toilets were mostly locked by their owners and most of them charge a fee for use (Figures 5, 6).

*“This is a picture of my latrine which I do not use. Many people here do not have toilets. I have no money to build a better one as the current one is a pit latrine which is difficult for me to use and it is in a bad shape-it needs repair. I ask myself of what use it is as I never use it and I always fear for the children who play around could fall into the latrine and that is very dangerous. I plead with the community to build us toilets that can accommodate all of us…”* (IDI, Older adult, Korogocho, F).

Participants also noted that that it was difficult for older adults to use the pit latrine, since most of them are not able to squat.

### Access to essential services and relief

Participants in Korogocho narrated the challenges created by lack of access to toilets, especially at night. The participants noted that houses in Korogocho were mostly built without toilets hence most residents strained to access toilet facilities resulting in the use of paper bags to relieve themselves, especially at night. Available toilets were mostly locked to avoid bad usage. In most cases, the owners of the toilets charged a fee for one to utilize the toilet. This poor infrastructure created vulnerabilities for older adults, and disabled persons, who faced mobility challenges to reach toilets and were at risk of violence if using them at night.

*“The smell, then again, you might not have a toilet, you use a bag. In the morning, you throw the bag into the drain. If all this blocks the drain outside my house, I will definitely get sick. …People do not have toilets here. Very few have toilets… Some people will be merciful and allow you to use their toilets or you pay 10 shillings for use. Or you have to go all the way to the bushes near the river to relieve yourself. What else can one do? There are no toilets”* (Older female IDI participant, Korogocho).

Older adults with health challenges struggled to access to health services and reported feelings of being left behind, in favor of youth and women.

*“Old is usually supposed to be gold… However, that is not the case in this community. No one treasures our knowledge. At every place you go, they say women and youth should have a priority. We end up isolated and we are going through difficult circumstances”* (IDI, Older adult, Korogocho, M).

For child-headed households, while different groups of children in various circumstances were identified as vulnerable and marginalized, including the boy child, girls with underage pregnancy and children with disability, children who headed households were identified as the most vulnerable and marginalized. Our participants reported that children heading households and orphans faced severe challenges of malnutrition, lack of education, and were restricted from accessing basic services that require identification documents as most did not have a caregiver to take the responsibility. Children heading households had to fend for their families at the expense of education, even when education was subsidized by the government (21).

*“Child headed households are marginalized, when there is registration of people to receive relief food, they are usually excluded because they lack identification card…child headed households have children marginalized from education services and are often restricted from accessing basic services”* (FGD, Parents of Adolescent, Korogocho, M).

**Figure 7:**
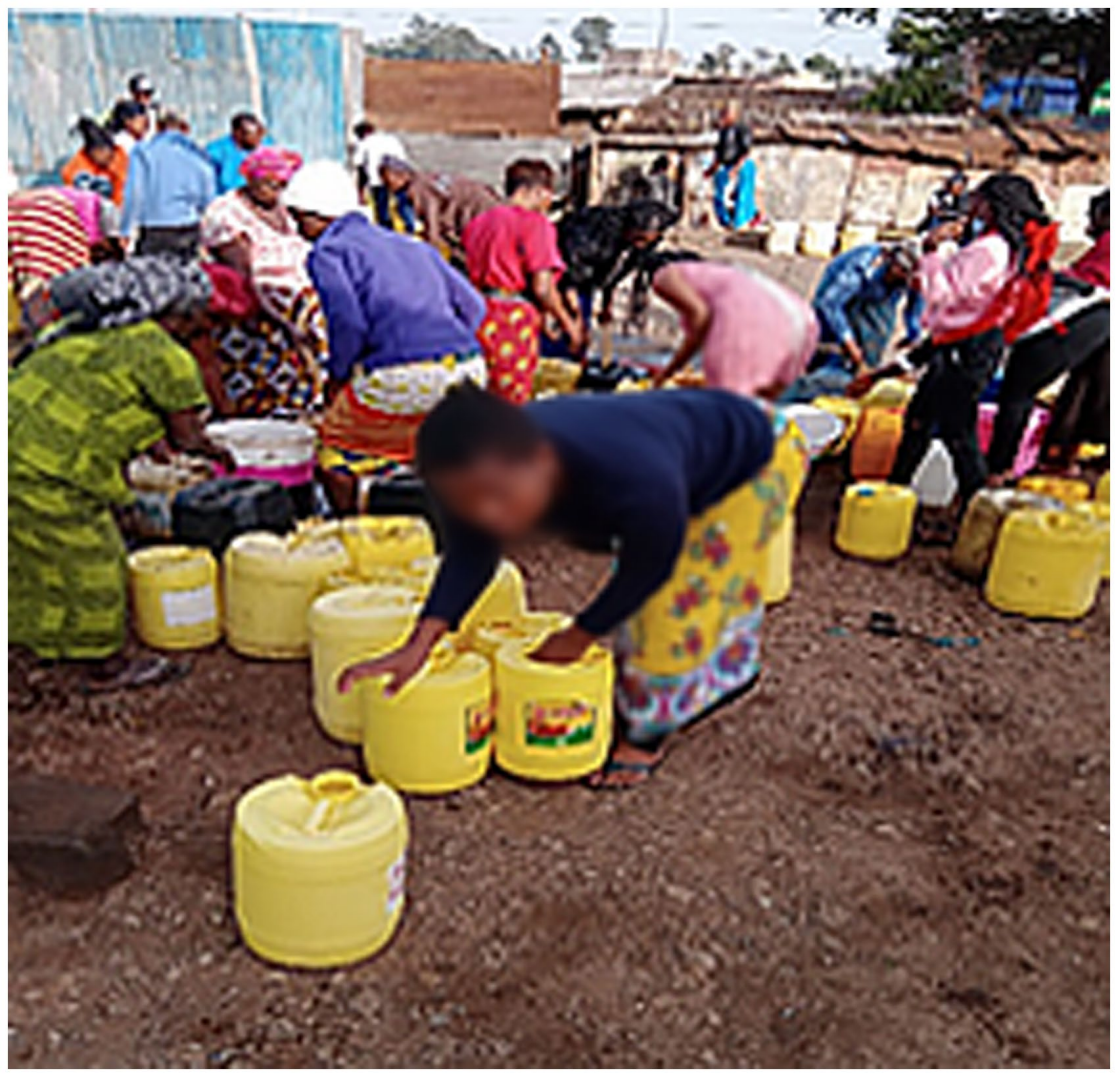
Access to water is a challenge for older adults living in informal settlements.

In a context of intense competition for available resources and services, older adults have lower capacity to physically “scramble” for access, including donations, for example the free water that was donated during the COVID-19 pandemic, as illustrated in Figure 7.

“*Mostly we {Older adult} cannot scramble like others; we step aside not unless someone is merciful to help us… so in many cases, we do not benefit from handouts/donations*” (IDI, Older adult, Viwandani, F).

*“When you go to the health clinic, you are not given a priority like other groups; they think you should be in school and not in the health facility seeking for child care services…to be sincere, we are going through difficult circumstances, people refer us as children with children and when there is relief food or water, we are not lucky to access… when in the health facility, it is the same thing… I usually try my level best not to go to public facilities like health center or even to think of going back to school”* (IDI, Child-head of household, Korogocho, F).

## Discussion of findings

Our focus on urban informal settlements in Nairobi is justified not only for spatially typifying inequity and deprivation but also by evidence that health is affected by factors arising from the shared physical and social environment, which have effects beyond those of poverty alone (10).

The methodological strength of using CBPR approaches is demonstrated by the contribution in accessing deep and rich data on marginalized and vulnerable sub-populations in neglected and poor urban informal settlements. Our study demonstrates that social mapping, governance diaries and photo voice are valuable and insightful CBPR approaches for engaging communities and exploring their marginalization and vulnerability. Each of the three CBPR methodologies enabled a nuanced understanding of the complex multiple dimensions of marginalization and vulnerability in the informal settlements, as well as the strength and complementarity of these methodologies.

The social mapping methodology enabled identification of the particularly vulnerable and marginalized groups within the complex heterogeneous settlements. The governance diaries led us to in-depth understanding of the perceptions of the particularly vulnerable and marginalized informal settlement residents regarding the various people and institutions with the power to influence health and wellbeing through time. As active participants on how to improve their living conditions, they identified the governance actors in their communities that can be built upon to enhance social accountability for equity in health service provision.

Our approach adds value to the existing global research landscape through a focus on harnessing the capacities of the urban poor to collect, analyze, and communicate their experiences of intersecting inequities, wellbeing, health, and governance challenges. Partnering with co-researchers from study communities was critical in promoting community based research approaches and accessing the inner recesses of the communities, especially the particularly vulnerable and marginalized, and during the COVID-19 pandemic, which placed entry hindrances into the settlements for outsiders. Supporting accountability measures entails having access to the right information at the right time and the height of the pandemic was such a time when real time data was needed to mobilize for interventions and to assess progress and incremental changes, hence the CBPR approaches came to the rescue.

Social mapping and understanding of the demographics of informal settlements are critical in identifying the particularly marginalized and vulnerable groups but contribute to a nuanced understanding of challenges and specific forms of marginality and vulnerability, making targeted policies, pinpointed priority setting and interventions possible in a multi-faceted and heterogeneous and fluid setting of these settlements. The Governance Dairies approach results in a deep description of how marginality is caused by factors that vary across different social groups, but always a complex interplay of various aspects that exclude some people from access to health and wellbeing services. It is important to underscore that similar drivers of marginality were the root cause of poverty in all study sites and for all groups in the informal settlements. We find that all the particularly marginalized and vulnerable groups share similar and interconnected challenges in access to basic services, including health services and food, in hindrances in accessing governance actors, in accessing the right and timely information, facing the challenges of discrimination, in accessing quality services, and in dealing with misinformation. They also share similar priorities relating to education, health, water, sanitation and hygiene, which runs through the entire settlements. In sum, we conclude that often the needs may be related to these settlements being both off the official maps, being socio-spatially undocumented and overall lack of priority in service provision, which is then exacerbated by intersectionality of the particularly marginalized and vulnerable sub-groups. Similarly, a key factor that contributes to marginalization have been linked to low socio-economic status of the poor, who have neither voice nor political clout to influence decisions on how public resources are used or distributed. Consequently, w*e* find that authority was key for both influencing and promoting equitable/inequitable service provision. These authority structures were identified by the particularly marginalized and vulnerable groups as embedded in families, neighborhood organizations, local leaders, relevant ministries and external leaders like funders, community based organizations (CBOs) and non-government organizations (NGOs). In addressing their challenges, vulnerable and marginalized groups preferred informal structures and networks as the actors were accessible, available and dedicated to their work compared to the formal actors. However, they identified challenges related to informal structures including the lack of expertise and sustainability of services, underscoring the need for complementarity of formal and informal actors in service provision. While the goal is leaving no one behind, the solution of inequity may also involve leaving no stakeholder and service provider behind. Study participants highlighted the need for formal actors working closely with the community actors as a strategy that could help formal structures to understand the community better as formal structures were perceived to be distant from the community and consequently perceived as inefficient (46).

The participatory wellbeing and governance analysis and priority setting, using the photo-voice approach, confirm that vulnerability and marginalization were driven by individual experiences together with other community level factors that affected informal settlement residents of all categories. At individual levels for instance, experience of marginalization and interconnected vulnerability among PWDs was shaped by the nature of their disability. A deaf person’s experience of marginalization is mostly centered around unintentional exclusion from health care and other services due to for instance, the lack of sign language interpreters. This intersected with poverty and financial constraint manifested through lack of money to obtain adequate food and nutrition, which further impacted their health increasing their need for health care services. Hearing impairment or deaf status is not obvious to other people, especially those who have not interacted with the deaf person. This interacted with the built environment, inclusive of wide unmarked tarmac roads without pedestrian paths, which increases deaf persons’ vulnerability to road accidents. This impacted negatively on the mental health of those with hearing impediments as they would become anxious at the thought of leaving the homes to access shops, markets, or health facilities. On the other hand, one with physical disability among study participants experienced marginalization and vulnerability through exclusion from economic activities resulting from a perception that he would not be able to carry out the manual jobs that he sought as required. Disability (physical) intersected with his gender increasing marginalization due to social stigma directed at him, which in turn affects his mental health, pushing him to alcoholism as a coping strategy. Persons with disability perceived limited capacities coupled with the burden of care, impacted their mental health, resulting in more need for health care services. Poverty and financial constraints intersected with the burden of care for self and children had an impact on the participant’s health and well-being as he worried about providing for his children who were solely dependent on him.

At the community level, participants with different disabilities experienced marginalization through the poor housing characterized by semi-permanent walls and roofs that would let in rodents and water/sewerage during rainy seasons, poor drainage, and lack of toilets.

Governance diaries expanded our understanding of the perceptions of diverse marginalized informal settlement residents, especially regarding various governance actors, in terms of people and institutions with the power to influence health and wellbeing. The governance diaries highlighted the perspective that authority was key for influencing equity/inequity in service provision. Governance diaries allowed us to build long-term relationships and understand temporal aspects of governance as it relates to the marginalized groups identified in the social mapping exercise. Our participants identified the governance actors in their communities that partner with community members to enhance social accountability for equity in health and wellbeing service provision. The participants identified formal and informal governance actors and their related networks. Informal actors identified included family, neighbors, friends, community groups and community members. Formal actors on the other hand included government institutions, individuals and authorities that make policies and rules as well as their desired and possible networks. Informal actors who were providing support services for the vulnerable populations directly without using agencies were described as having direct networks, while the formal actors that had the potential of providing support services because they were offering the services in the community were described as having possible networks. There were formal actors who were identified by study participants to be of great influence in providing support services but were not felt nor accessible to marginalized and vulnerable groups. These groups were described as having desired networks. Notably, some actors were identified as having multiple networks, and in terms of addressing vulnerability and marginality moving forward, formal and informal actors and their networks were identified as having complementary roles that were beneficial to the particularly vulnerable and marginalized groups living and working in these informal settlements. As active participants in their daily grinds, study participants characterized vulnerability and marginalization severally, identified their exacerbating factors, and strongly expressed them in terms of challenges in access to basic services, discrimination when services are available, lack of quality services, inadequate information on basic services and governance actors, misinformation and difficulties in accessing governance actors. Finally, the photo voice approach enabled the recording and reflection on the day-to-day lived experiences of marginalization that affect the health and wellbeing of people with disability, older adults and children heading households.

The photo voice study, giving voice to the lived experiences of the three vulnerable categories of residents, will help inform pointed planning and quality service delivery. The following are areas of consideration as informed by the findings. There is a need for the full dissemination and implementation of Kenya’s persons with disability Act of 2003 as a measure to reduce social stigma against PWDs, increase their participation in economic activities and to fully realize their rights, especially the right of participation in decision making (47). Urban planning inclusive of building infrastructure needs to be cognizant of the needs of persons with disability including the construction of disability-friendly roads with restricted pedestrian paths. Most persons with disability especially those born with disability have faced multiple discrimination and subsequent denial of education and other opportunities, compounding their marginalization and vulnerability. Thus, there is need for special consideration of children born to persons with disability in the issue of bursaries and other interventions implemented at the local levels. There is a need to focus on all vulnerable children including children heading households who may have both or one parent. Social protection interventions such as cash transfers and public health care services through subsidized comprehensive public health insurance fund that may cater for the whole family need to also include children heading households as a vulnerable category. Child heads of households go through hardships that require counseling hence the need for psychosocial support delivered through community health structures where these children are known and can be easily identified and provided with psychosocial support services. There is a need for data on children heading households to facilitate prioritization for targeted public interventions such as school bursaries and other programs such as DREAMS (48). Building on the inter-connected community based participatory methodologies, it became clear that assessing what needs to be improved in an informal settlement, will require acknowledging local intelligence, wisdom and creativity to its form as opposed to the notion of chaos and grim. There is existing human capital, systems and strategies that local actors and planners need to acknowledge, adapt and build upon in urban policy, urban design and interventions, so as to promote equitable health and wellbeing. Examples of policies to build on include informal settlement upgrading policies (55), social assistance and protection programs (56), and school feeding programs (57), among others. Notwithstanding, current and often highly fragmented social structures/actors in informal settlements face different socio-economic, technical, and biophysical challenges that need to be formally addressed in order to deliver quality and sufficient services.

The key message out of our methodological approaches is that multiple and interconnected participatory methods that build on each other generate a more in-depth wider perspective of issues. This is consistent with advantages found by other research efforts in understanding and addressing urban risks in sub-Saharan Africa. According to Dodman et al., the range of hazards of events that can cause death, illness or injury, and impoverishment across urban Africa is a rationale for using a spectrum of methods to address such a spectrum of risks. The utility of mixed-methods approaches in building a stronger evidence base to provide a more solid base for planning and investment were demonstrated. As well as the importance of taking individual contexts into account, there are underlying methodological principles that can inform a range of related approaches to understanding urban risks and inform the breaking of the cycle of risk accumulation in sub-Saharan Africa (45).

## Conclusion

The social and governance mapping as well as the photo voice, conducted with separate groups, provided nuanced understanding of how different groups experience the communities differently. It was useful in understanding key social inequalities within the study communities and how these influence the health and wellbeing and access to services for vulnerable and marginalized informal settlement residents in Nairobi. Understanding these dynamics remains essential for Kenya in the renewed push to achieve the poverty reduction goals spelt out in the SDGs and Kenya’s Vision 2030. It also contributes to the continent-wide literature in the search for pathways to address the increasing concern of viability and sustainability of urban living especially for the particularly vulnerable urban poor and agendas to promote inclusive urbanization in the region. The outcomes provide many informal settlement specific evidence, which reiterates the need for the design and implementation of sub-group differentiated anti-poverty policies and interventions among the urban poor.

Beyond the deep experiences and narratives uncovered by the paper, the key point is the rationale the paper provided for using a combination of community based participatory approaches to address a spectrum of marginalization and vulnerability and the demonstration of the utility of different methodological approaches in identifying vulnerable groups and the nature of vulnerability and marginality. It describes studies undertaken on similar subjects using multiple approaches to gather empirical data, in order to build a stronger evidence base and provide a more solid base for planning and interventions. It highlights the importance of joining methodological forces that speaks to specific contexts and different layers of complexities in different urban informal settlements.

### Positionality statement

The research team was multi-disciplinary and comprised members with two dominant epistemological standpoints: positivist and constructivist. During the protocol development, we had consensus to adopt a post-positivist approach to the entire study. This meant we concurrently implemented both qualitative and quantitative participatory methods in the study sites to respond to our research questions (49, 50, 58). The application of participatory quantitative methods will be published in separate articles. Throughout the study, we convened reflexity sessions with researchers to assess how their positions (as outsiders to informal settlements), values and beliefs influenced their interactions with community co-researchers. We also conducted quarterly Ripple Effect Mapping sessions with community co-researchers to gain insights into how they perceived the effect of the CBPR approaches. Using appreciative inquiry approaches during the REM sessions, we explored how community co-researchers perceived their interactions with researchers.

### Limitations

The study was conducted in two informal settlements, however, the diversities in the informal settlements provide a clear picture on exploration of voices and challenges and could be similar to other informal settlements in Nairobi and outside Nairobi. Data collection happened at the peak of COVID-19 and it might not be a true reflection of the settlements in the absence of the COVID-19 outbreak. However, informal settlements are always prone to outbreaks ranging from fire, floods, clashes and other diseases (i.e., non-COVID-19) outbreak. Lastly due to the focus of this study on the methodological contribution, we only presented top-tier results, as such other detailed findings are documented in other reports and manuscripts.

### Ethical considerations

Ethical clearance to conduct this study was obtained from the AMREF Ethics & Scientific Review Committee (ESRC; AMREF-ESRC P747-2019). National clearance to conduct the study was also obtained from National Commission for Science, Technology, and Innovation (NACOSTI)(NACOSTI/P/20/7726). All participants and co-researchers provided written informed consent to participate in this study. We also obtained written informed consent for the publication of any potentially identifiable images or data included in this article. We re-consented participants to obtain permission from them to publish their photos in this article. We provided comprehensive training on safeguarding to all co-researchers and participants in accordance with the guidelines established by some of the co-authors of this article (11). Within our safeguarding guidelines, we determined that the use of smartphones by co-researchers and participants in Korogocho and Viwandani would introduce an additional risk of theft. We closely monitored the smartphones to ensure data security and regular data backups. At the outset of our study, we conducted training on the ethics of photography for all participants and co-researchers, offering continuous guidance on the ethical aspects of photo voice throughout the study duration. We also trained co-researchers on how to obtain written informed consent from participants. Importantly, we did not encounter any safeguarding issues and ethical dilemmas during the course of our research.

## Data availability statement

The raw data supporting the conclusions of this article will be made available by the authors, without undue reservation.

## Ethics statement

The studies involving humans were approved by AMREF Ethics, Scientific and Research Committee. The studies were conducted in accordance with the local legislation and institutional requirements. Written informed consent for participation in this study was provided by the participants’ legal guardians/next of kin. Written informed consent was obtained from the individual(s), and minor(s)’ legal guardian/next of kin, for the publication of any potentially identifiable images or data included in this article.

## Author contributions

RK, ST, RS, CK, LOT, IC, NM, LOK, and BM: study conception and design. IC, IN, LOK, and NM: data collection. RK, CK, IC, IN, LOK, NM, and BM: analysis and interpretation of results. RK, ST, RS, CK, KO, LOT, IN, IC, RT, RS, LD, JK, NM, and BM: draft manuscript preparation. All authors reviewed, contributed to the article, and approved the submitted version.
